# Reactivity in the human retinal microvasculature measured during acute gas breathing provocations

**DOI:** 10.1038/s41598-017-02344-5

**Published:** 2017-05-18

**Authors:** Angelina Duan, Phillip A. Bedggood, Andrew B. Metha, Bang V. Bui

**Affiliations:** Department of Optometry & Vision Sciences, The University of Melbourne, Victoria, 3010 Australia

## Abstract

Although changes in vessel diameter following gas perturbation have been documented in retinal arterioles and venules, these responses have yet to be quantified in the smallest vessels of the human retina. Here, using *in vivo* adaptive optics, we imaged 3–25 µm diameter vessels of the human inner retinal circulation and monitored the effects of altered gas-breathing conditions. During isocapnic hyperoxia, definite constrictions were seen in 51% of vessel segments (mean ± SD for pre-capillary arterioles −9.5 ± 3.0%; capillaries −11.8 ± 3.3%; post-capillary venules −6.3 ± 2.8%); these are comparable with responses previously reported in larger vessels. During isoxic hypercapnia, definite dilations were seen in 47% of vessel segments (mean ± SD for pre-capillary arterioles +9.8 ± 1.5%; capillaries +13.7 ± 3.8%; post-capillary venules +7.5 ± 4.2%); these are proportionally greater than responses previously reported in larger vessels. The magnitude of these proportional changes implies that the capillary beds themselves play an important role in the retinal response to changes in carbon dioxide levels. Interestingly, the distribution of microvascular responses shown here differs from our previously reported responses to flicker stimulation, suggesting differences in the way blood supply is coordinated following gas perturbation and altered neural activity.

## Introduction

The demands of neuronal activity far exceed any energy or oxygen stores in neural tissue^1, 2^, meaning that constant supply from the circulation is crucial for normal neural health and function. Understanding the hemodynamic response of the smallest vessels in the circulation has been of recent interest in neural tissue research. This is in part due to their proximity to neurons, which is thought to enable a tight coupling between neurons and their blood supply, forming the neurovascular unit^3^. Control of blood flow at this level of the circulation provides the greatest spatial resolution for precise delivery of nutrients and oxygen where required. As such, local changes in blood supply during neural activity are believed to form the signal for blood-oxygen-level-dependent functional magnetic resonance imaging^4^. Characterizing the hemodynamic response of neural vasculature following functional stimulation is therefore important for understanding this widely used technique.

In addition to functional reactivity, neural vasculature can adapt to regulate oxygen supply and carbon dioxide removal. For instance, the human inner retinal circulation is able to adjust in response to changes in the partial pressure of oxygen (PaO_2_) and carbon dioxide (PaCO_2_)^5, 6^. Inhaling altered gas mixtures produces a change in the systemic levels of PaO_2_ and PaCO_2_ in healthy awake human participants^7^. Increasing levels of PaO_2_ (hyperoxia) constricts larger retinal arterioles^8, 9^ and venules^10–13^. Conversely, increasing PaCO_2_ (hypercapnia) dilates those vessels^14, 15^. Impaired vessel reactivity to gas breathing is implicated in neurovascular disease pathogenesis, with a reduced response to gas perturbation seen in human participants with hypertension^16^, progressive open angle glaucoma^17^, and type 2 diabetes mellitus^18^.

While changes in vessel caliber in human neural vasculature during altered gas breathing have been documented in health and disease, studies to date have measured responses only in blood vessels with baseline diameters >85 µm^8–13, 16–19^. Changes in capillary vessel diameter (<8 µm) have been reported in the cerebral vasculature of anaesthetized animals following changes in the levels of carbon dioxide^20–22^, and in cerebral and retinal vasculature following increased oxygen levels^23^. However, it is still unknown as to whether and to what extent similar changes occur in the human retinal microvasculature.

Here, we used adaptive optics to image the smallest vessels of the human inner retinal vasculature (baseline diameter <25 µm) following gas breathing perturbations, to determine whether these vessels undergo caliber changes similar in magnitude to those reported previously in larger retinal vessels. Since oxygen and carbon dioxide are thought to drive changes in vessel diameter via different pathways^24–26^, we quantified the small vessel response to hyperoxia and hypercapnia independently.

We also compare the magnitude and distribution of proportional microvascular responses seen in this study to our previously reported responses, in similar retinal regions, where a 1.25° spot was flickered on the retina to produce localized increased neural activity^27^. As the gas perturbation used in this study has been delivered systemically and should not specifically alter neural activity, differences in the distribution of response across the vascular network response may shed further light on the notion of neurovascular coupling in the microvasculature.

## Results

### Gas Breathing Conditions

The average end-tidal gas pressures for each breathing condition across all 3 participants are summarized in Table 1. Under isocapnic hyperoxia there was a significant increase in end tidal PaO_2_, but no change in PaCO_2_. A similar level of control was achieved during isoxic hypercapnia with a significant elevation of PaCO_2_, with PaO_2_ remaining at baseline levels.

**Table 1:** Average P_ET_O_2_ and P_ET_CO_2_ values and SD for each breathing condition.

Breathing Condition	P_ET_O_2_ (mmHg)	P_ET_CO_2_ (mmHg)
Baseline	116.3 ± 9.3	35.4 ± 3.5
Isocapnic Hyperoxia	482.2 ± 30.42	34.7 ± 3.4
Isoxic Hypercapnia	118.1 ± 8.7	44.4 ± 2.2

Data shown includes every measurement taken at the time of imaging for each participant.

### Baseline Vessel Description

**Figure 1:**
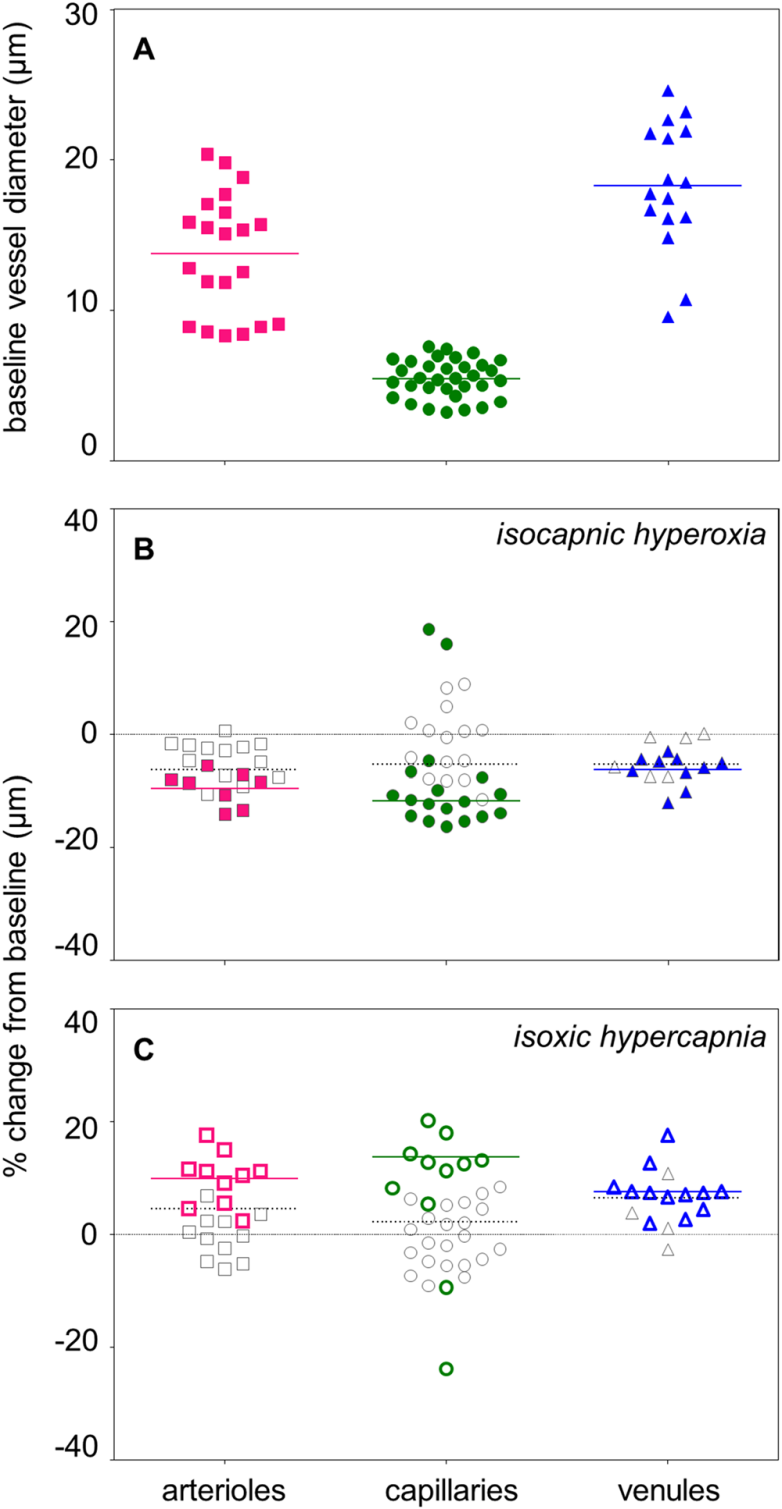
(**A**) Illustrates baseline vessel diameter of all 70 segments analysed (21 were from pre-capillary arterioles, 33 from capillaries and 16 from post-capillary venules). The average vessel width for each vessel type is indicated with a colored solid line (pre-capillary arterioles = 13.8 µm, capillaries 5.5 µm and post-capillary venules = 18.3 µm). (**B**) Average responses of all 70 segments to isocapnic hyperoxia. (**C**) Average responses of all 70 segments to isoxic hypercapnia. For both (**B**,**C**), colored symbols represent definite responses. Segments classified as non-responders (grey symbols) are included for completeness. The average proportional change of “responders” (colored symbols) is given in a colored solid line, and the average proportional change for all segments of that vessel type is marked with a grey dotted line. Note that the two capillary segments which responded in a direction opposite to expected have not been included in the calculation of average response for “responders”.

70 vessel segments, with baseline vessel widths 3.2–24.6 µm, were analysed from 12 regions of interest across the 3 participants (Fig. 1A). There were 21 segments taken from pre-capillary arterioles, 33 segments taken from capillaries and 16 segments taken from post-capillary venules (Fig. 1A). The average vessel widths for each vessel type are summarized in Table 2.

**Table 2:** Summary of mean ± SD (coefficient of variation) baseline vessel diameter and mean ± SD proportional changes in vessel diameter measured in all segments during systemic gas perturbations.

Vessel type	Baseline vessel diameter (mean ± SD)	Vessel reactivity (mean ± SD)	
		Isocapnic hyperoxia	Isoxic hypercapnia
Pre-capillary arterioles	13.8 ± 4.0 µm (0.29)	−6.3 ± 4.0%	+4.4 ± 6.7%
Capillaries	5.5 ± 1.3 µm (0.23)	−5.4 ± 9.0%	+2.2 ± 9.2%
Post-capillary venules	18.3 ± 4.3 µm (0.24)	−5.2 ± 3.3%	+6.5 ± 4.8%

### Overall Vessel Response

**Figure 2:**
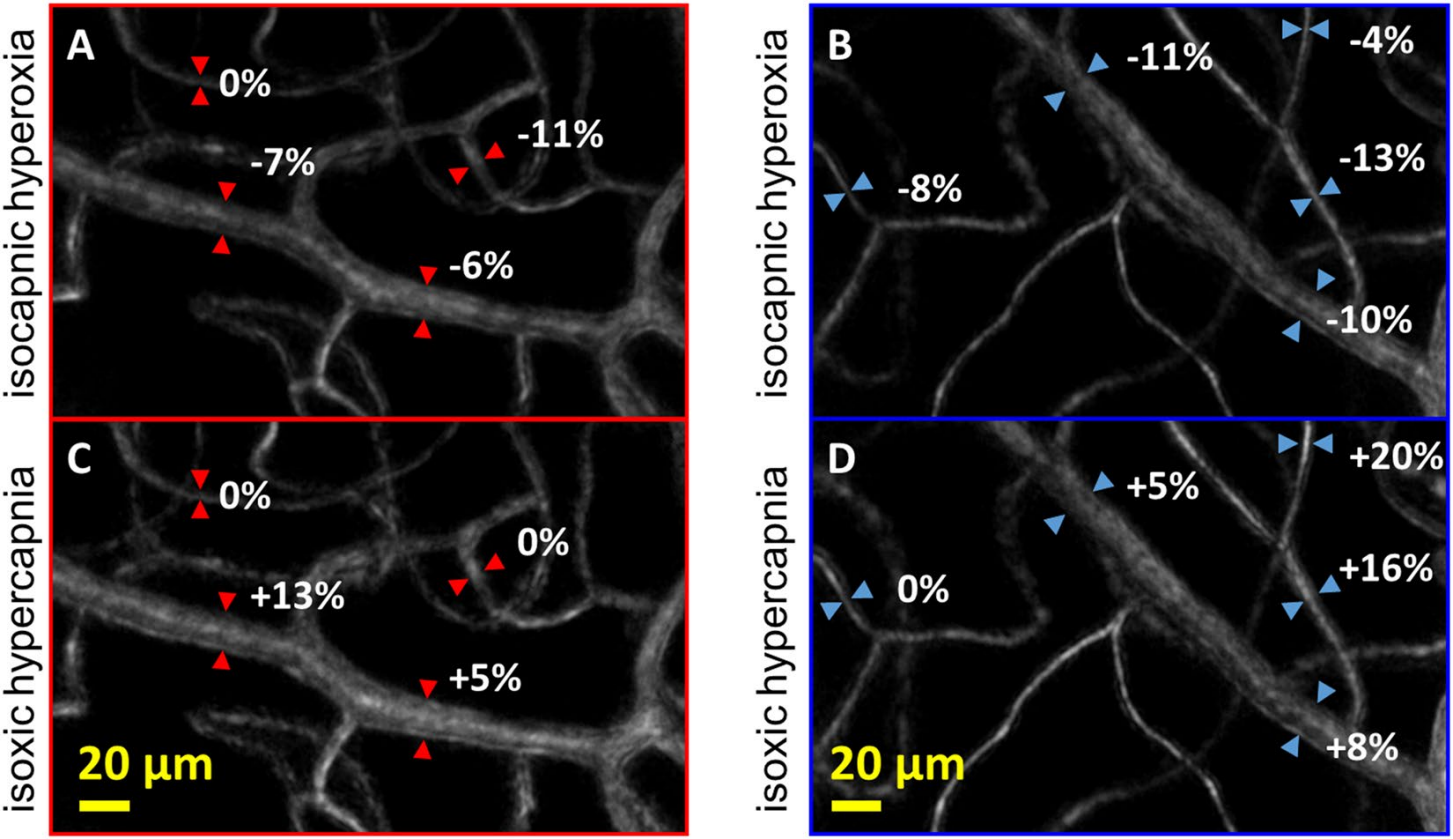
Examples of a region of interest containing a “major” arteriole (red) and a “major” venule (blue), with vessel responses following isocapnic hyperoxia (**A**,**B**) and isoxic hypercapnia (**C**,**D**) marked.

The average response along the entire segment of each vessel is shown in Fig. 1B and C and summarized in Table 2. Of the 70 segments analysed, most vessels constricted following isocapnic hyperoxia (Fig. 1B). Examples of constriction are shown in a region of interest containing a “major” arteriole (Fig. 2A) and a “major” venule (Fig. 2B). Conversely, most vessels dilated following isoxic hypercapnia (Fig. 1C). Examples of dilation are shown in a “major” arteriole region (Fig. 2C) and a “major” venule region (Fig. 2D). These responses were not seen consistently across any vessel type for either gas breathing condition; there were instances of vessels not responding (Fig. 2), or responding in the opposite direction to expected (Fig. 1B and C). The variance in vessel responses was similar between subjects, as were the mean response size and direction (Supplementary Table 1 and Supplementary Figure 1).

**Figure 3:**
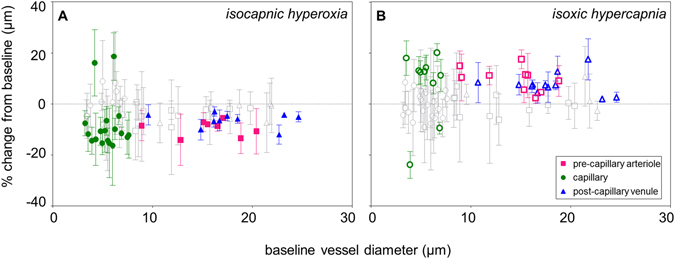
The proportional change from all 70 vessel segments analysed following isocapnic hyperoxia (**A**) and isoxic hypercapnia (**B**) with colored data points representing vessel segments demonstrating a definite response (whose 2 SEM error bars do not encompass zero), and grey data points representing vessel segments showing no measurable response (whose 2 SEM error bars encompass zero). There was a generalized constriction (**A**) following isocapnic hyperoxia and a generalized dilation (**B**) following isoxic hypercapnia. Proportional responses did not vary as a function of vessel size.

There was no relationship between vessel response and vessel size when all 70 vessel segments were analysed (Fig. 3A and B) under isocapnic hyperoxia (p = 0.9) or isoxic hypercapnia (p = 0.2).

### Definite Responses

Some of the 70 segments did not demonstrate a measurable average response (grey data points in Figs 1B and C and 3A and B). In these “non-responder” segments, there was either zero proportional change, or the response did not fall outside of baseline vessel diameter variability (±2 SEM error bars encompass zero in Fig. 3A and B). During isocapnic hyperoxia, a definite response was measured in 38% of pre-capillary arterioles, 55% of capillaries and 63% of post-capillary venules (Fig. 1B). During isoxic hypercapnia, a definite response was measured in 48% of pre-capillary arterioles, 33% of capillaries and 75% of post-capillary venules (Fig. 1C). The mean ± SD proportional responses of “responders” are given in Table 3. As noted above for the overall vessel response, it was observed that response variability and mean response were also similar across subjects in this subset of vessels classed as definite responders. (see Supplementary Table 2 and Supplementary Figure 2).

**Table 3:** Summary of mean ± SD proportional changes in vessel diameter measured in “responders” during systemic gas perturbations.

Vessel type	Vessel reactivity (mean ± SD)	
	Isocapnic hyperoxia	Isoxic hypercapnia
Pre-capillary arterioles	−9.5 ± 3.0%	+9.8 ± 4.7%
Capillaries (expected)	−11.8 ± 3.3%	+13.7 ± 3.8%
Post-capillary venules	−6.3 ± 2.8%	+7.5 ± 4.2%

Note that the two capillary segments that responded in the opposite direction to expected have not been included.

### Flicker vs. Gas Perturbation

**Figure 4:**
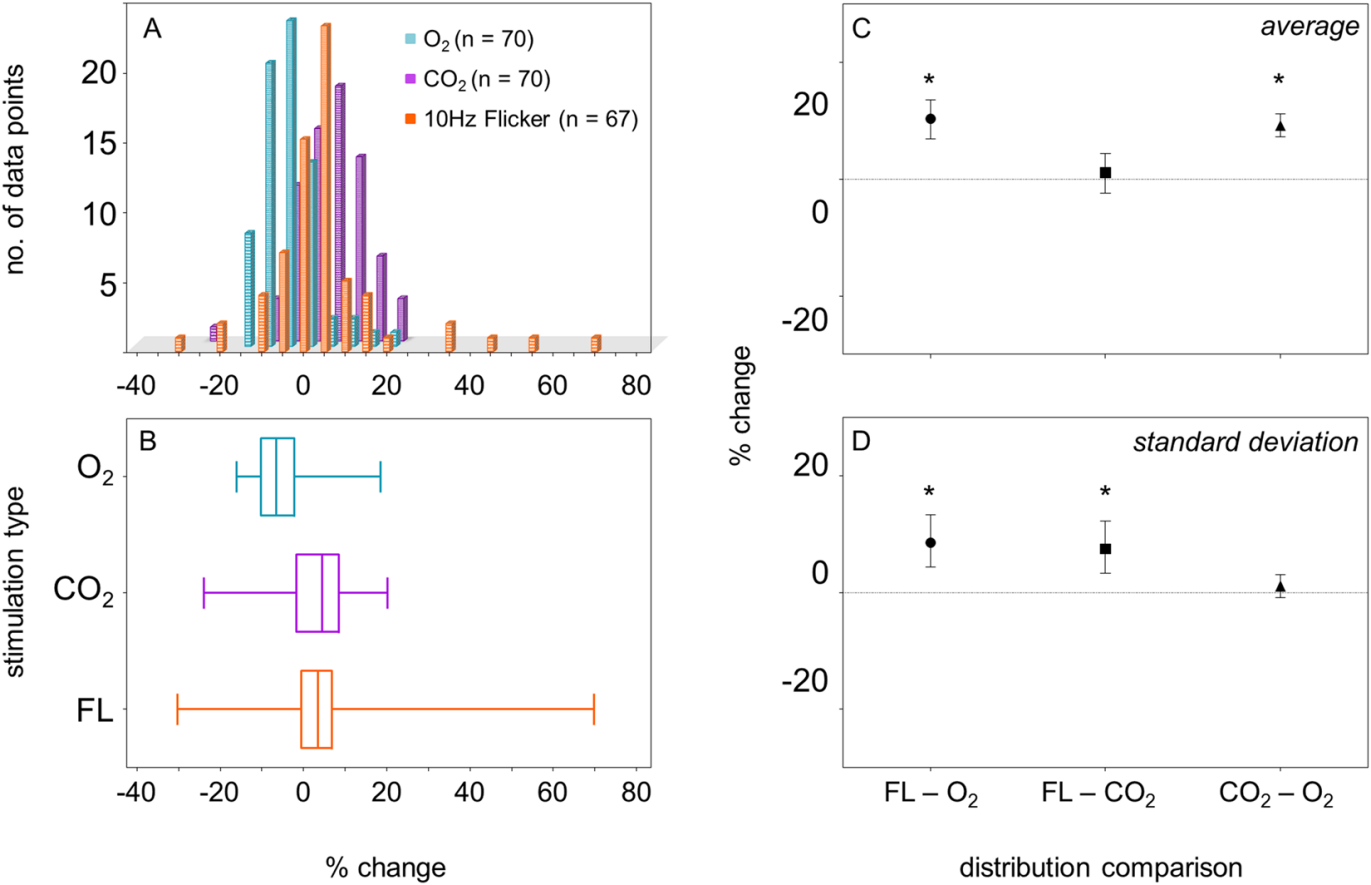
(**A**) Frequency histogram of proportional change for all 70 segments following isocapnic hyperoxia (blue, referenced as O_2_) or isoxic hypercapnia (purple, referenced as CO_2_), and for all 67 segments analysed following 10 Hz flicker (orange, referenced as 10 Hz FL). (**B**) Box plot showing the maximum, minimum, median and interquartile range of the distribution for all 70 segments following isocapnic hyperoxia, isoxic hypercapnia and all 67 segments following flicker. (**C**) Difference in mean and (**D**) standard deviation of all possible combinations for flicker (FL), isocapnic hyperoxia (O_2_) and isoxic hypercapnia (CO_2_) conditions with bootstrapped 95% confidence intervals. Asterisks denote statistical significance (p < 0.05).

The distribution of all average responses seen following isocapnic hyperoxia, isoxic hypercapnia and flicker are shown in Fig. 4A and B. As expected, vessels constricted on average following isocapnic hyperoxia (−5.4%), but dilated on average following isoxic hypercapnia (+3.8%) and flickering light (+5.0%) (Fig. 4A). During flicker stimulation, there was a greater spread of vessel responses (Fig. 4B). By bootstrapping the distribution of vessel responses, it was possible to compare differences in mean and spread (standard deviation) between the three conditions. Both flicker and isoxic hypercapnia produced similar mean vasodilation (Fig. 4C). However, in comparison to the gas breathing conditions, flickering light stimulation resulted in a distribution of vessel responses with significantly wider spread (Fig. 4D).

## Discussion

Here, using adaptive optics imaging, we show for the first time that the inner human retinal microcirculation with baseline diameter <25 µm demonstrates a measurable change in diameter to increased PaO_2_ and PaCO_2_ (Fig. 1B and C).

Changes in either oxygen or carbon dioxide levels can modulate vessel diameter via several potential pathways^21, 28^. As such, although there is a large body of gas perturbation literature, we will compare our results with controlled isocapnic and isoxic gas breathing protocols. Studies of human retinal vessels (baseline diameters >85 µm) challenged with a similar isocapnic hyperoxia protocol to ours report constrictions ranging from −7% to −14%^8, 9, 29–32^. Here, we show that a comparable definite constriction ranging from −6% to −10% can be measured in vessels with baseline diameter <25 µm (Table 3). Isoxic hypercapnic challenge that raises PaCO_2_ to the same level as our protocol has been shown to generate vasodilation ranging from +3% to +6% in retinal vessels (diameters >85 µm)^19, 33, 34^. Our averaged dilation was approximately double, ranging from +8% to +14% (Table 3).

It is important to note that our subjects were under gas stimulation for 2.5 minutes, which is somewhat shorter than other studies^8, 19, 29, 33^. Stimulus durations as short as 3 minutes have been shown to produce robust changes to vessel diameter^8, 13, 29^. Whilst vessel reactivity to oxygen stimulus is thought to be largest after 5 minutes^35^, the vessel response to carbon dioxide is reportedly faster, occurring after 3 minutes^15^. Given these previous studies, it is possible that our shorter protocol has led, if anything, to an underestimation of the maximal vasoconstriction in response to isocapnic hyperoxia.

The average pre-capillary arteriolar response, measured in those vessel segments which demonstrated a definite response (colored symbols, Fig. 1B and C), was a constriction of −10% during isocapnic hyperoxia (Table 3). This is comparable to the −7% to −12% constriction reported for larger retinal arterioles under similar gas breathing conditions^29^. However, these same vessel segments showed a dilation of +10% during isoxic hypercapnia (Table 3), which is greater than the +3% to +4% responses previously reported in larger retinal arterioles^19, 33^. As red blood cell diameter is often equal to or greater than the vessel lumen in the smallest vessels of the circulation^36, 37^, a change in diameter in these vessels is thought to have a greater effect on red blood cell transit time than classically predicted by Poiseuille’s Law^38^. Given this, our findings suggest that pre-capillary arterioles may show a more passive response to isocapnic hyperoxia, but may play a larger role in regulating blood flow during isoxic hypercapnia.

In post-capillary venule segments that demonstrated a definite response (colored symbols, Figs 1B and C and 3A and B), the constriction of −6% during isocapnic hyperoxia (Table 3) was smaller than the −14% constriction previously reported in larger venules^30^, whilst the 8% dilation following isoxic hypercapnia (Table 3) was comparable to the +6% reported in larger venules^34^.

In this study, we demonstrate for the first time that human retinal capillary diameter changes in response to altered systemic gas conditions *in vivo* (Fig. 1B and C). Tissue slices from animal brain show that capillaries can change in diameter following gas perturbations despite not having a layer of smooth muscle^20, 21, 28^. There is also evidence that the resting state of contractile pericytes found on retinal capillaries may be modulated by changes in oxygen and carbon dioxide perfusion levels. For instance, it is thought that pericytes are relaxed in the presence of nitric oxide released by endothelial cells^39^, and, as oxygen can degrade nitric oxide, an increase in oxygen can produce constriction of pericytes^40^. Additionally, bovine retinal capillary pericytes have demonstrated the ability to contract and relax during increased and decreased carbon dioxide perfusion, respectively. This was thought to be driven the concomitant change in pH that occurs with altering carbon dioxide^41^. However, in this study, the proportional change exhibited by capillaries were similar to those seen in pre- and post- capillary vessels (Table 3), so it is possible that there is no active process occurring at the level of the capillaries in the human retina *in vivo*.

Of the 70 segments analysed, 49% of segments during isocapnic hyperoxia and 53% of segments during isoxic hypercapnia did not display a significant response (outside the 95% confidence interval of the baseline). Due to the nature of data obtained from flood-based adaptive optics imaging, images from multiple runs needed to be averaged to produce a single, final image for analysis. Baseline vessel diameter is expected to vary over time due to dynamic systemic parameters such as fluctuations in blood pressure and the cardiac cycle. There are also run to run variations in image quality caused by a combination of factors including fixation instability between imaging sequences. Thus, it is unclear whether some segments truly showed no change in diameter, or just changed by an amount less than the noise due to variations in image quality and baseline vascular physiology. Nevertheless, of the vessel segments classified as “responders”, the direction and magnitude of vessel responses during both gas perturbations generally agreed with the nature of responses previously reported in the literature.

A flow redistribution model has been proposed which suggests that the heterogeneity seen in the blood flow supplying neural tissue at rest exists to allow for modification in tissue perfusion during neural activity^42, 43^. From this model, tissue oxygenation can be increased by decreasing heterogeneity in erythrocyte transit times across the vascular bed^42^. Given the heterogeneity already present in the capillary network supplying neural tissue, it is expected that a wider range of vessel response directions and magnitudes is required to achieve this^44, 45^.

In our previous work, we flickered a small 1.25° patch of light at a frequency of 10 Hz and found that a wide range of vessel response directions and magnitudes occurred, supportive of the flow redistribution model. Here, in the same vasculature, we altered systemic oxygen and carbon dioxide levels, which is expected to provide a more generalized stimulus to flow regulation. Indeed, both isoxic hypercapnia and local 10 Hz flicker stimuli for vasodilation produced similar mean vasodilation (Fig. 4C)^27^, and, whilst some capillaries responded in an unexpected direction following both gas breathing conditions, these were few (Fig. 1B and C). Vessel responses following local neural stimulation from flickering light showed a distribution with significantly wider spread compared with gas challenge (Fig. 4D). Again, this is consistent with the idea that changes to vessel diameter in the microvessels may reflect changes in the larger upstream feeder vessels, especially in response to isocapnic hyperoxia. Conversely, with local flickering stimuli, there is less change to upstream vessels and a more local redistribution of flow at the level of the microvasculature, possibly to maximize metabolic exchange.

Whilst some care should be taken in generalizing for the population, we believe that our observations are robust for the following reasons. Firstly, for all 3 participants, it was consistently noted that variability in vessel responses occurred both within a region of interest, and between regions of interest. This type of pattern is inconsistent with run-to-run variations that might occur due to defocus of the image, or pulse-related changes in regional blood flow. Secondly, all 3 participants showed the same pattern of response to both gas conditions, both in their overall vessel responses, (Supplementary Figure 1) and in their subset of vessels that were definite responders (Supplementary Figure 2).

Our results demonstrate that the vessel caliber changes measured in larger inner retinal vessels also exist at the level of the retinal microvasculature. The similarity in proportional response across pre-capillary arterioles, capillaries and post-capillary venules under isocapnic hyperoxia suggests a systematic redistribution of blood flow in response to increased PaO_2_. As the dilation response across pre-capillary arterioles, capillaries and post-capillary venules under isoxic hypercapnia is greater than previously reported in the larger retinal vessels, the smallest vessels of the retina may play a greater role in the response to increased PaCO_2_. Remarkably, the response profile of the microvasculature to local flicker stimulation is entirely different to that of either gas breathing condition, supporting the notion of highly specific flow redistribution during neural activity.

## Materials and Methods

### Subjects

Three healthy subjects (2 males and 1 female, aged 27–43 years) with mean refractive spherical error <4 D and astigmatic error <2 DC, optically clear media and stable fixation critical for small vessel imaging experiments participated. This study was approved by the Human Research Ethics Committee of the University of Melbourne and all testing conformed to the tenets of the Declaration of Helsinki. All participants provided written informed consent. Participants were free from systemic and ocular diseases, not taking any medications, had no history of ocular surgery, were within normal BMI with normal cardiovascular status, and had no family history of cardiovascular disease. Participants were asked to refrain from caffeine or strenuous exercise 12 hours prior to data collection^46^. The left eye of each participant was dilated 20 minutes prior to being imaged with one drop of 0.5% tropicamide (Alcon, USA) with additional top-ups as necessary.

### Gas Breathing Perturbation

The RespirAct^TM^ (Thornhill Research, Inc., Toronto, Canada) calculates the required mix and flow of specific source gases to generate consistent changes in the end-tidal partial pressure of oxygen (P_ET_O_2_) and carbon dioxide (P_ET_CO_2_) in participants^7^ via computer controlled sequential gas delivery into a re-breathing circuit (Hi-Ox, Viasys Healthcare, Yorba Linda, California, USA)^33^. The re-breathing circuit covers the mouth and nose of the participant and is sealed to the face with adhesive tape (Tegaderm, 3 M Health Care, St Paul, MN, USA). The PaCO_2_ and PaO_2_ was detected by measuring P_ET_CO_2_ and P_ET_O_2_^47^ which were monitored throughout all gas breathing conditions.

Vessel diameter changes occur within 1 minute following increased oxygen inhalation^35^ and carbon dioxide inhalation^15^. In our study, room air was initially delivered into the re-breathing circuit during a 2-minute period of acclimation. Once P_ET_O_2_ and P_ET_CO_2_ levels had stabilized following acclimation, the participant’s average baseline P_ET_O_2_ and P_ET_CO_2_ were measured over 10 normal breaths to create the baseline target for P_ET_O_2_ and P_ET_CO_2_.

**Figure 5:**
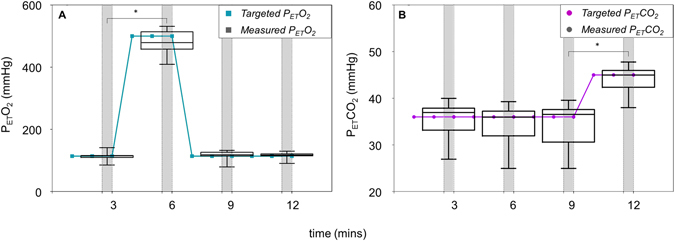
Changes in end-tidal partial pressure of oxygen (P_ET_O_2_) and carbon dioxide (P_ET_CO_2_) during the gas breathing sequence. (**A**) Target P_ET_O_2_ (green line) and measured P_ET_O_2_ (black box and whisker plots), along with (**B**) target P_ET_CO_2_ (purple line) and measured P_ET_CO_2_ (black box and whisker plots) from all 3 participants across the 12-minute imaging sequence. Whiskers show minimum and maximum data, and boxes show median and interquartile range. Breathing conditions were as follows: baseline (0–3 mins), isocapnic hyperoxia (3–6 mins), return to baseline (6–9 mins), and isoxic hypercapnia (9–12 mins). A period of 30 secs (grey bars) was allowed for image acquisition during each breathing condition.

For isocapnic hyperoxia breathing conditions, target P_ET_O_2_ was 500 mmHg and P_ET_CO_2_ was clamped at each participant’s baseline P_ET_CO_2_ (Fig. 5A and B)^9^. For isoxic hypercapnia breathing conditions, target P_ET_O_2_ was clamped at each participant’s baseline P_ET_O_2_ (≈110 mmHg) and target P_ET_CO_2_ was a 9% increase (≈10 mmHg) from the participant’s baseline P_ET_CO_2_ (≈35 mmHg) (Fig. 5A and B)^14^. Gas breathing perturbations were interleaved with baseline breathing conditions to control for time related changes in retinal haemodynamics (Fig. 5A and B).

### Flicker Perturbation

The results from the gas breathing perturbation were compared to previously collected flicker perturbation results in the same retinal microvasculature. The full protocol has been published^27^ but in brief, subjects were asked to fixate on a static, dimly illuminated grid (detailed below) for 20 seconds before baseline images were collected. The same fixation was maintained for 20 seconds whilst the light used for imaging (1.25° size, bandwidth 593 ± 25 nm, full width at half maximum) was flickered at 10 Hz on low power (50% duty cycle with 100% contrast, square wave, 4.7 µW when turned on) before post-flicker images were collected.

### Imaging Region

**Figure 6:**
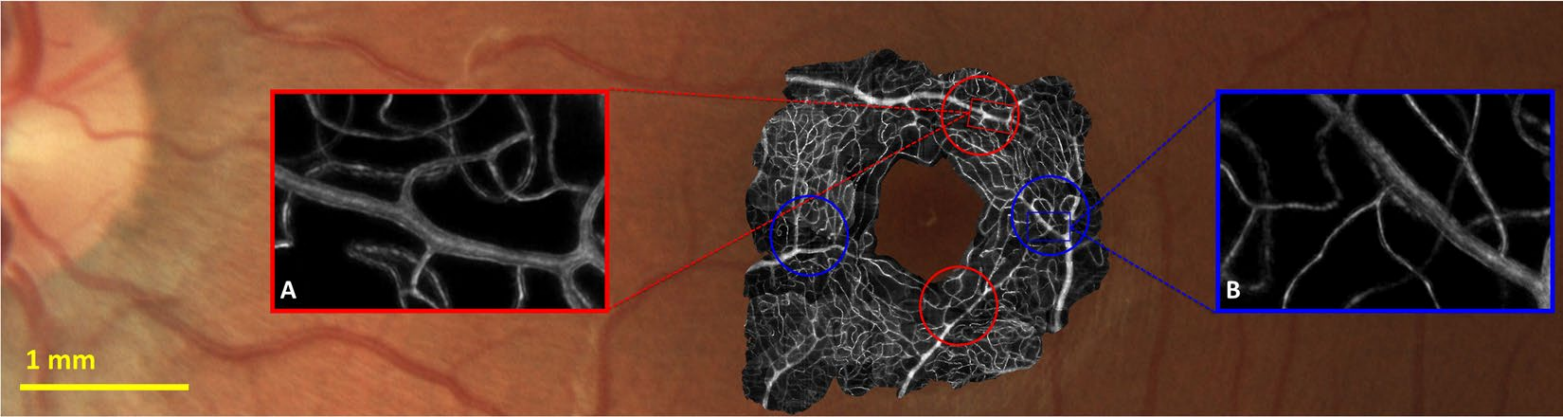
Standard retinal image showing inner retinal vasculature of one participant (color photo) overlaid with motion contrast adaptive optics images (black and white) demonstrating the size and location of vessels analysed in this study. Inset outlined in red (**A**) shows an example region of interest containing a “major” arteriole; inset outlined in blue (**B**) shows an example region of interest containing a “major” venule. Both correspond to the regions of interest illustrated in Fig. 2.

The 4 regions of interest selected from the retinal microvascular network of each participant had centers located 2–2.6° (580–750 µm) radially from the foveal center (Fig. 6), providing 12 regions in total. Each region of interest was 1.25° (360 µm) in diameter and included either a “major” arteriole (Fig. 6A) or venule (Fig. 6B) and their associated branches (8–25 µm) and capillaries (3–8 µm). Participants were asked to fixate on a labelled 5 × 5° fixation grid (black markings 1° apart, printed on white paper, back-illuminated with white LED light source passed through diffuser) to place the region of interest into the field of view and aid fixation. With the room lights off, the effective luminance viewed through the system was 1.75 cd/m^2^.

### Adaptive Optics

A flood-illumination adaptive optics ophthalmoscope described previously was used to acquire images^48^. Briefly, light from a superluminescent diode source (835 nm) was focused onto the retina and re-imaged by lenslets of a Shack-Hartmann wavefront sensor (f = 24mm, pitch 0.4 mm). A 97-channel deformable mirror (Alpao, Montbonnot St. Martin, France) driven by custom Matlab software (Mathworks, Natick, MA) corrected the measured ocular wavefront aberration in real time (20 fps).

### Imaging Sequence

Each image acquisition of 80 frames occurred at 200 fps (400 ms acquisition time). When root mean square wavefront error decreased below 0.06 µm over a 7 mm pupil, image acquisition was manually triggered, sending a transistor-transistor logic (TTL) pulse to a logic controller that also waited for a trigger synchronized to the participant’s cardiac cycle (described below). Once both signals were received, the camera (Neo sCMOS; Andor Technology PLC, Belfast, UK) was triggered. At the beginning of each 2.5 ms frame exposure, the camera sent its own TTL pulse to drive the imaging light source [8 W supercontinuum laser, 0.33 mW at 593 ± 25 nm full-width at half maximum (Fianium Ltd., Southampton, UK)]. To reduce coherence (and image speckle), the imaging light was passed through 32 m of 0.37 NA, 200 µm core diameter, step-index optical fiber (Thorlabs, Newton, NJ). Imaging with 593 nm light through a 7 mm pupil results in diffraction-limited resolution better than 0.9 µm; the camera plane magnification is set so that the image is sampled at a spatial scale of 0.5 µm per pixel.

### Cardiac Cycle Synchronization

Vessel diameter changes are expected over our 400 ms acquisition period, due to the cardiac cycle^49^. To reduce physiological variability in vessel diameter, data were collected at the same phase of the cardiac cycle for all participants by monitoring the participant’s pulse with an analogue optical finger pulse monitor (Model #1260, Sunrom technologies, Gujarat, India). Once the participant had acclimatized to the gas breathing condition, a systolic peak was detected and the camera was programmed to begin capturing images at the time point equal to 67% of the participant’s previous inter-beat interval. Image sequences were approved if the actual systolic peak during the acquisition period was accurate to within 10% of this goal; otherwise, the data was discarded and the image acquisition run was repeated.

### Image Processing

Each image acquisition of 80 frames underwent a series of image processing steps including background subtraction, flat-fielding and co-registration to produce a single average (bright-field) and motion-contrast image for baseline, isocapnic hyperoxia and isoxic hypercapnia breathing conditions (Fig. 2)^50, 51^. To improve edge detection, each frame was resampled before registration and averaging to generate a final image that is 5 times larger, so that the spatial extent of each pixel is 0.1 µm. This interpolation can be made reliably as the images are captured with 12-bit pixel resolution coupled with low read noise.

### Image Analysis

An investigator masked to the experimental condition was asked to select all visible vessel segments of sufficient image quality for analysis resulting in a total of 70 present in all experimental conditions. The centerline of each vessel segment of interest was manually traced in Photoshop CS6 (Adobe, USA) and the vessel trace was then applied across all conditions and to the “2 × SEM images” described below. Traces were then used to straighten and center the segment against its background^27^, resulting in a single, centered and straightened representation of the segment of interest, with any nearby vessels appearing to be distorted due to their varying curvatures and orientations with respect to the segment of interest. These distortions do not affect the diameter measurement of the vessel segment of interest.

### Vessel Edge Detection

Whilst a Gaussian model is commonly fit to the intensity profile of a vessel to determine vessel^52^, the intensity profile of many of the smaller vessels did not resemble the Gaussian shape observed in conventional imaging of larger retinal vessels. Instead, we developed a custom edge detection algorithm written in Matlab to quantify the diameter at each point along straightened vessel segments which were aligned horizontally in an image array. This has been described in detail elsewhere^27^ but in brief, after manually masking vessel branches or crossings on vessel segments of interest, an edge estimate is made by locating the adjacent pixel pair with greatest proportional change in intensity. Constraints were then added to the algorithm to reduce the impact of noise, and the estimated vessel edges were plotted for manual assessment of accuracy.

If the edge detection for any vessel segment was deemed unsatisfactory for any of the five masked images (baseline, isocapnic hyperoxia, isoxic hypercapnia or two “SEM images” defined below), all data for that vessel segment was removed from further analysis. This occurred in 39 of the 109 initially selected segments, demonstrative of the noisy nature of capillary image data. A large contributor to poor signal-to-noise resulted from eye movements whilst attempting to maintain fixation. As the regions of interest were chosen so that the “major” arteriole or venule was centered in the field, the segments with lower signal-to-noise ratio were often capillaries. In addition to this, naturally some portions of the blood vessels dived into and out of the plane of focus selected for imaging, producing inconsistent focus and image quality for those segments.

Once the vessel edges were located, the distance between the two edges was calculated for each column as the measure for vessel diameter, and the identity of the vessels were decoded to print the results.

### Vessel Classification

We defined capillaries as vessels with baseline diameter ≤8 µm, as single-file flow of erythrocytes can be seen in our movie data in vessels of this diameter and smaller. This is similar to criterion used in histological studies of the inner human retinal circulation^53^. All other vessels were identified as either pre-capillary arterioles or post-capillary venules based on the direction of blood flow visible from imaging at 200 fps.

### Statistics

#### Quantification of vessel diameter

The change in diameter along the length of each vessel segment was averaged to describe the overall proportional change in diameter for each vessel type. Linear regression on this overall change was used to determine whether any relationship between vessel response and vessel size existed.

#### Estimation of uncertainty

To determine whether there were any residual effects on vessel diameter resulting from prior gas breathing conditions, we analyzed baseline diameter for runs collected immediately (2.5 minutes) following isocapnic hyperoxia, and compared this to baseline diameter for runs immediately following isoxic hypercapnia. Sufficient data (more than 3 images for each condition) existed for 49 vessel segments which were included for analysis (see Supplementary Figure 4). A two-tailed paired t-test showed no significant difference in baseline vessel diameters following each breathing condition (p = 0.14), so all baseline data across the imaging sequence was combined to improve signal to noise ratio from the low contrast images derived from this system without contrast dye. Specifically, the data from 80 frames collected across 400 ms was averaged for each imaging sequence to create 10 baseline breathing images and 10 altered breathing images. For each breathing condition, we then averaged the data to create a single image. Whilst this improves the signal-to-noise ratio for each image, averaging data from different time points during the imaging sequence introduces variability as both image quality and baseline vessel diameter can change over time due to systemic factors. Traditional statistical methods to estimate variability would require the analysis to be performed on a subset of the images, but we found the resulting reduction in signal-to-noise ratio too great to perform meaningful analysis. Instead, we devised the concept of “±2 SEM images” where the standard error of the mean intensity for each pixel across all imaging sequences for the baseline condition is calculated and doubled. Secondly, this value is added to or subtracted from the averaged image to create two images representing the ±2 SEM variability, respectively. Both “2 × SEM images” are segmented and analysed with the edge detection algorithm as per the averaged images, by the same blinded investigator. The “2 × SEM image” (positive or negative) that provided the largest difference from baseline diameter was conservatively used to produce ±2 SEM confidence intervals for the measure of baseline diameter (see Supplementary Figure 3). During isocapnic hyperoxia or isoxic hypercapnia, any vessel segments exhibiting a change in diameter outside of the ±2 SEM confidence interval for their respective baseline measurement were classified as definite “responders”.

#### Comparison of vessel response distributions

The difference in the mean and standard deviation between the stimulus conditions of isocapnic hyperoxia, isoxic hypercapnia and 10 Hz flicker were bootstrapped 10,000 times to generate a 95% confidence interval to determine significance with a pseudo-alpha level of 0.05.

## Electronic supplementary material

Supplementary Information
