# Supplementary material for: Reactivity in the human retinal microvasculature measured during acute gas breathing provocations
Source: Sci Rep. 2017 May 18;7:2113. doi: 10.1038/s41598-017-02344-5 (PMC5437020; doi:10.1038/s41598-017-02344-5)
Supplement: Supplementary file 1 — Supplementary Information [file 41598_2017_2344_MOESM1_ESM.doc]

Title:

Reactivity in the human retinal microvasculature measured during acute gas breathing provocations

Authors:

Angelina Duan^1^, Dr. Phillip A Bedggood^1^, Associate Professor Andrew B. Metha^1^, Associate Professor Bang V. Bui^1^*

^1^Department of Optometry & Vision Sciences, The University of Melbourne, Victoria, 3010, Australia

*Corresponding author

Email address: [*bvb@unimelb.edu.au*](mailto:bvb@unimelb.edu.au)

Phone: +61 3 83447006

Fax: +61 3 93497498

**Consistency of response to gas challenge amongst individuals**

As noted in the manuscript (see Results section, paragraph ‘Overall Vessel Response’), variability in the vessel response size across the vascular bed was seen following gas breathing conditions. This variability (illustrated for 2 regions of interest in 1 subject in Figure 2) was seen within all regions of interest for all individuals.

However, it is important to rule out the possibility that the measured change in vessel diameter may arise from difference between individuals. Firstly, to confirm that the variability for all measured vessel responses was comparable between individuals, a 1-way ANOVA was conducted on all vessel responses from each subject to show there was no statistically significant difference in either the mean or the variance in vessel responses between subjects (Supplementary Table 1 and Supplementary Figure 1).

| **Breathing condition** | **1-way ANOVA statistic** | **Bartlett’s Test of Equal Variances** |
| --- | --- | --- |
| Isocapnic hyperoxia | F (2,67) = 0.8, p = 0.5 | p = 0.5 |
| Isoxic hypercapnia | F (2,67) = 0.5, p = 0.6 | p = 0.3 |

***Supplementary Table 1:*** *1-way ANOVA results showing no significant difference in vessel response variability for all 70 vessel segments between all 3 participants.*


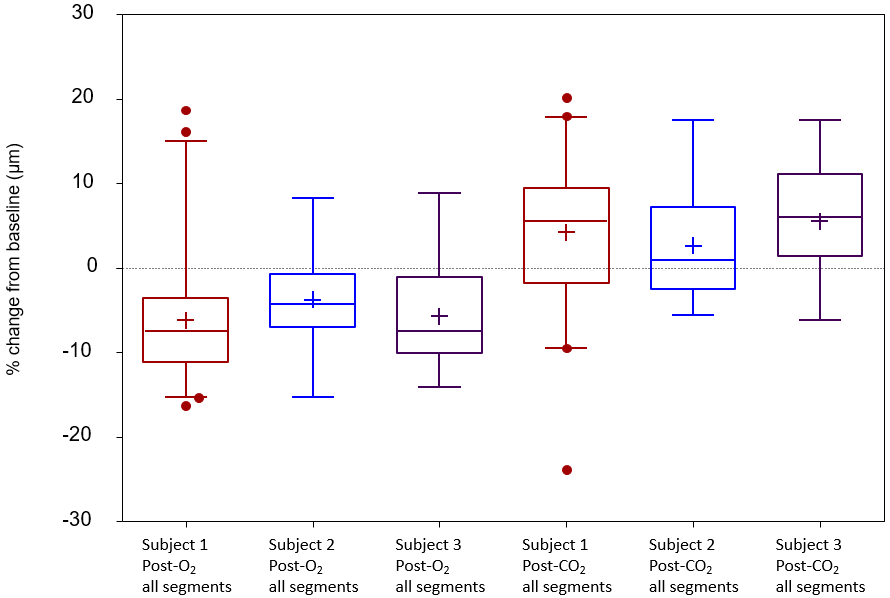


***Supplementary Figure 1:*** *Summary of proportional responses from all segments from each participant following isocapnic hyperoxia and isoxic hypercapnia. This data includes segments that did not respond (had 0% change), or segments where vessel response did not fall outside baseline vessel variabiltiy. Box plots show 5^th^ to 95^th^ percentile, median and interquartile range of proportional vessel response, crosses indicate the average. Outliers are indicated as symbols.*

Furthermore, to check that the variability of definite responses was similar among the three subjects, a 1-way ANOVA was conducted on all definite vessel responses from each subject to show there was no statistically significant difference in either the mean or the variance in definite vessel responses between subjects (Supplementary Table 2 and Supplementary Figure 2).

| **Breathing condition** | **1-way ANOVA statistic** | **Bartlett’s Test of Equal Variances** |
| --- | --- | --- |
| Isocapnic hyperoxia | F (2,33) = 0.3, p = 0.8 | p = 0.1 |
| Isoxic hypercapnia | F (2,30) = 0.4, p = 0.7 | p = 0.1 |

***Supplementary Table 2:*** *1-way ANOVA results showing no significant difference in response variability for all definite responding segments from each of the 3 participants.*


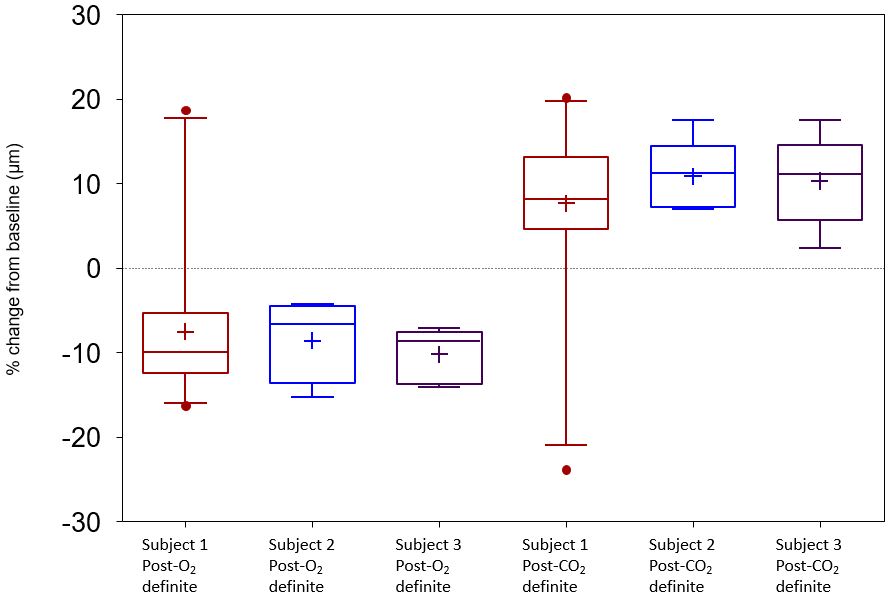


*Supplementary Figure 2: Summary of only the definite proportional responses for each participant following isocapnic hyperoxia and isoxic hypercapnia. Box plots show 5^th^ to 95^th^ percentile, median and interquartile range of proportional vessel response, crosses indicate the average. Outliers are indicated as symbols.*

**Establishing baseline vessel diameter variability**

Traditional measures of variability, such as the coefficient of variations, may be less useful for our purpose for two reasons. Firstly, a wide range of vessel sizes and types were intentionally selected from all levels of the vascular tree present in each region of interest (illustrated in Figure 2). Secondly, to create the baseline image for analysis, we averaged across 10 baseline imaging sequences (200 frames for each of the baseline, isocapnic hyperoxia, baseline and isoxic hypercapnia conditions illustrated in Figure 5).. Thus, as we averaged information across time, it is expected that variations in baseline vessel diameter will occur irrespective of stimulation as a result of spontaneous vasomotion (Braun, Linsenmeier et al. 1992, Chen, Patel et al. 1994) and blood pressure (Riva, Grunwald et al. 1986), as well as changes in image quality across the field of view resulting from the participant’s tear film and fixation stability. This creates the need to estimate baseline vessel measurement variability for each vessel segment analysed. Our approach was to establish robust confidence limits around the baseline diameter for each individual vessel segment. The 2xSEM estimate for baseline vessel diameter is given for each individual vessel (error bars, Figure 3) which can be used to give an idea of the repeatability of the diameter measurement for each vessel segment at baseline.

To achieve this, we use the 800 frames to calculate the standard error of the mean intensity for each pixel in the baseline image. This value is doubled (i.e. 2xSEM) then added or subtracted from each pixel intensity, to generate two images that respectively show ± 2 SEM variability. Each of the ± 2 SEM images are then analysed with the same edge detection algorithm used on the averaged image. To be conservative, the 2xSEM image with the largest different from baseline diameter was then used to establish the confidence intervals for each vessel segment. This returns a measure of variability for each vessel segment at baseline as shown in Supplementary Figure 3, which is the proportional difference between baseline diameter and the most conservative 2xSEM diameter (either the +2 SEM or -2 SEM image) for all 70 segments in the data set.


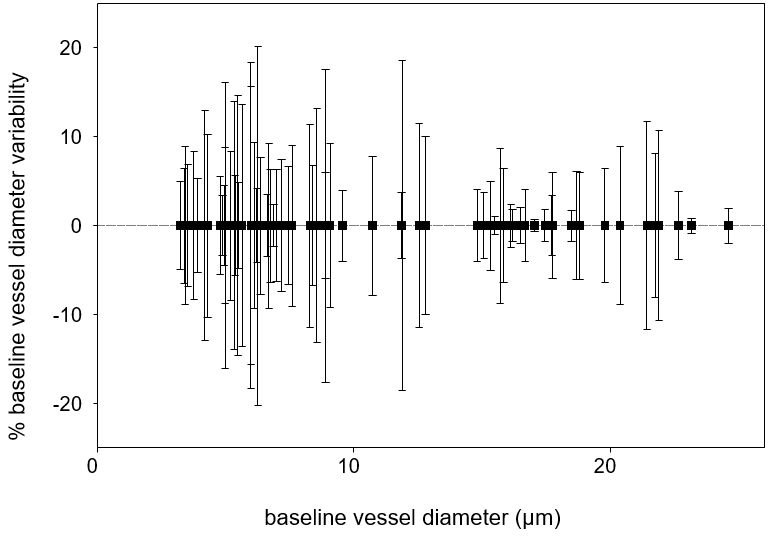


***Supplementary Figure 3:*** *Proportional variability in baseline vessel diameter for each of the 70 segments included in the analysis. These values were generated from the largest proportional difference established by adding (+2 SEM) or subtracting (-2 SEM) 2 times SEM to each pixel in the baseline image. This conservative approach provides a measure of internal variability for each vessel segment. For a vessel response to be considered a definite responder to gas challenge, the proportional response had to be greater than the estimated baseline variability for that vessel segment.*

**Ruling out interactions between gas conditions**

As baseline and gas perturbation conditions were interleaved, it is important to ensure that gas breathing stimuli did not influence baseline vessel diameter, or the subsequent response to gas perturbation.

To confirm that there was no significant difference in the baseline vessel diameter following each gas breathing perturbation (i.e., that enough time had been given for vessels to return to pre-stimulus diameters), a random subset of the 70 data segments analysed were selected and re-analysed to show baseline vessel diameter following isocapnic hyperoxia and isoxic hypercapnia. A two-tailed paired t-test for these 49 vessel segments showed no significant difference in vessel diameter post-O_2_ and post-CO_2_ (p = 0.14). The absolute vessel diameters (µm) post-stimuli are shown in Supplementary Figure 4.


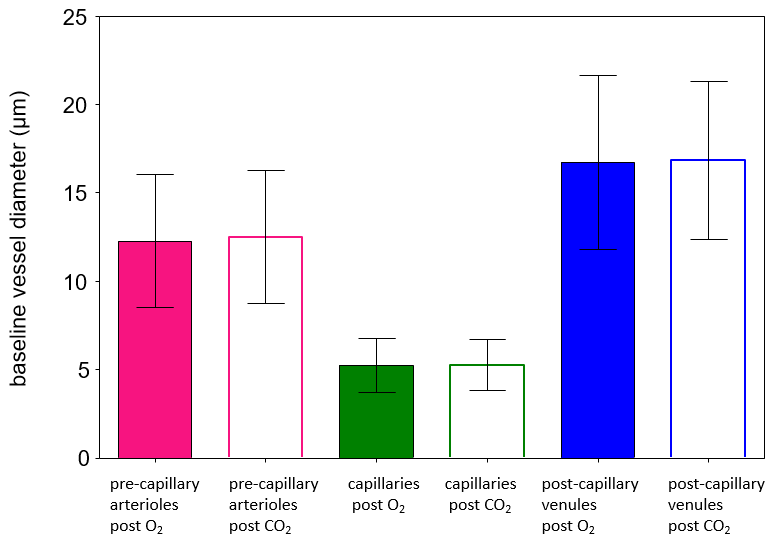


***Supplementary Figure 4:*** Mean ± SD baseline diameter for a subset of pre-capillary arterioles (n = 12), capillaries (n = 27) and post-capillary venules (n = 10) from all 3 participants measured 2.5 minutes after isocapnic hyperoxia (filled bars) and after isoxic hypercapnia (unfilled bars). There was no significant difference between the two baseline diameter measurements from this subset, so all baselines were combined for analysis in the final data set.

**References**

Braun, R. D., R. A. Linsenmeier and C. M. Yancey (1992). "Spontaneous fluctuations in oxygen tension in the cat retina." Microvasc Res **44**(1): 73-84.

Chen, H. C., V. Patel, J. Wiek, S. M. Rassam and E. M. Kohner (1994). "Vessel diameter changes during the cardiac cycle." Eye (Lond) **8 ( Pt 1)**: 97-103.

Riva, C. E., J. E. Grunwald and B. L. Petrig (1986). "Autoregulation of human retinal blood flow. An investigation with laser Doppler velocimetry." Invest Ophthalmol Vis Sci **27**(12): 1706-1712.
